# Non-Lytic, Actin-Based Exit of Intracellular Parasites from *C. elegans* Intestinal Cells

**DOI:** 10.1371/journal.ppat.1002227

**Published:** 2011-09-15

**Authors:** Kathleen A. Estes, Suzannah C. Szumowski, Emily R. Troemel

**Affiliations:** Division of Biological Sciences, University of California, San Diego, La Jolla, California, United States of America; University of Birmingham, United Kingdom

## Abstract

The intestine is a common site for invasion by intracellular pathogens, but little is known about how pathogens restructure and exit intestinal cells *in vivo*. The natural microsporidian parasite *N. parisii* invades intestinal cells of the nematode *C. elegans*, progresses through its life cycle, and then exits cells in a transmissible spore form. Here we show that *N. parisii* causes rearrangements of host actin inside intestinal cells as part of a novel parasite exit strategy. First, we show that *N. parisii* infection causes ectopic localization of the normally apical-restricted actin to the basolateral side of intestinal cells, where it often forms network-like structures. Soon after this actin relocalization, we find that gaps appear in the terminal web, a conserved cytoskeletal structure that could present a barrier to exit. Reducing actin expression creates terminal web gaps in the absence of infection, suggesting that infection-induced actin relocalization triggers gap formation. We show that terminal web gaps form at a distinct stage of infection, precisely timed to precede spore exit, and that all contagious animals exhibit gaps. Interestingly, we find that while perturbations in actin can create these gaps, actin is not required for infection progression or spore formation, but actin is required for spore exit. Finally, we show that despite large numbers of spores exiting intestinal cells, this exit does not cause cell lysis. These results provide insight into parasite manipulation of the host cytoskeleton and non-lytic escape from intestinal cells *in vivo*.

## Author Summary

The intestine is a common site for invasion by pathogens, but little is known about how pathogens exit out of live intestinal cells in order to spread and propagate. One group of parasites that often invades the intestine is the microsporidia, which comprise a phylum of over 1200 fungal-like species that can cause disease in humans, as well as in agriculturally significant organisms such as fish, silkworm and honey bee. Here, we investigated a natural microsporidian infection in live intestinal cells of the roundworm *C. elegans*. We discovered a novel exit strategy used by microsporidia to restructure the cytoskeleton of intestinal cells, involving relocalization of actin and reorganization of a structure called the terminal web, which may be a barrier to exit. In addition, we found that despite large numbers of parasites exiting out of intestinal cells, this process does not cause cells to burst. Our findings indicate that microsporidia, which are completely dependent on their hosts for replication, have evolved a regulated and non-damaging mechanism of exit that shares similarities with strategies used by evolutionarily distant bacterial pathogens. This study provides new insights into the methods by which pathogens restructure live intestinal cells to facilitate their spread and propagation.

## Introduction

Intracellular pathogens have a life cycle that includes three major steps: invasion of the host cell, replication, and exit out of the host cell. While the question of how pathogens invade cells has been intensively studied, much less is known about how pathogens exit cells, although this process appears to be highly regulated [1]. Even when pathogen exit causes lysis of host cells, this lysis is often due not simply to mechanical stress, but is part of a regulated process controlled by the pathogen. For example, the intracellular bacterium *Chlamydia* uses a cysteine protease to lyse host cells at the proper time as a mechanism of escape [2]. In addition to lytic escape, there are also less destructive modes of pathogen exit. One example of non-lytic pathogen exit comes from the Gram-positive bacterium *Listeria*, which polymerizes host actin to force its way into neighboring cells, which then engulf the bacterium to allow cell-to-cell spread [3]. Another example of non-lytic pathogen exit involving actin is used by *Mycobacterium* [4], [5]. In contrast to *Listeria*, *Mycobacterium* appears to break through the host plasma membrane as it is exiting, and the membrane reseals behind the pathogen such that the host cell is not lysed. Acquiring a better understanding of the mechanisms of pathogen exit *in vivo* could lead to better treatments in a variety of settings, since the process of exit is critical for the propagation and spread of intracellular pathogens of all types.

Many intracellular pathogens invade their host and progress through infection in the intestine. However, most studies of pathogen exit have been performed in tissue culture cells or single-celled hosts, such as the studies described above. Unfortunately, these model hosts lack the connectivity, differentiated structures, and polarity of intact intestinal cells. The *Caenorhabditis elegans* intestinal tract provides an excellent system to study intestinal pathogens as it is composed of 20 epithelial cells that share many morphological properties with human intestinal epithelial cells [6]. In both humans and worms, the intestine contains polarized epithelial cells decorated with apical, finger-like microvilli anchored into a cytoskeletal structure called the terminal web, which is composed of actin and intermediate filaments. Because *C. elegans* intestinal cells share these structural similarities with human intestinal epithelial cells and because nematodes are transparent, *C. elegans* provides a very useful whole-animal model for study of host/pathogen interactions in intestinal epithelial cells [7], [8].

Recently, we described the first natural intracellular pathogen of *C. elegans* and found that it defines a new species of microsporidia [9], [10]. Microsporidia are obligate intracellular parasites that can infect virtually all animal phyla, as well as a few protists [11], [12], [13]. These parasites comprise a phylum that is either part of the fungal kingdom or is a sister group to the fungi [14], [15], [16], [17]. The Microsporidia phylum contains over 1200 species, 14 of which can cause infection in humans. These infections most commonly afflict AIDS patients and other immunocompromised patients where they can cause persistent and lethal diarrhea [18]. Because so little is known about these microbes and few treatments are available, they have been deemed priority pathogens by the U.S. National Institutes of Health. Microsporidia are also considered microbial contaminants of concern by the U.S. Environmental Protection Agency and can plague agriculturally relevant organisms. For example, microsporidia have been responsible for the collapse of fisheries and have also been implicated in honey bee colony collapse disorder [19], [20], [21], [22]. We named the *C. elegans*-infecting microsporidia species *Nematocida parisii*, or nematode-killer from Paris, since it was first isolated from a French wild-caught nematode and it eventually kills its host.

**Figure 1:**
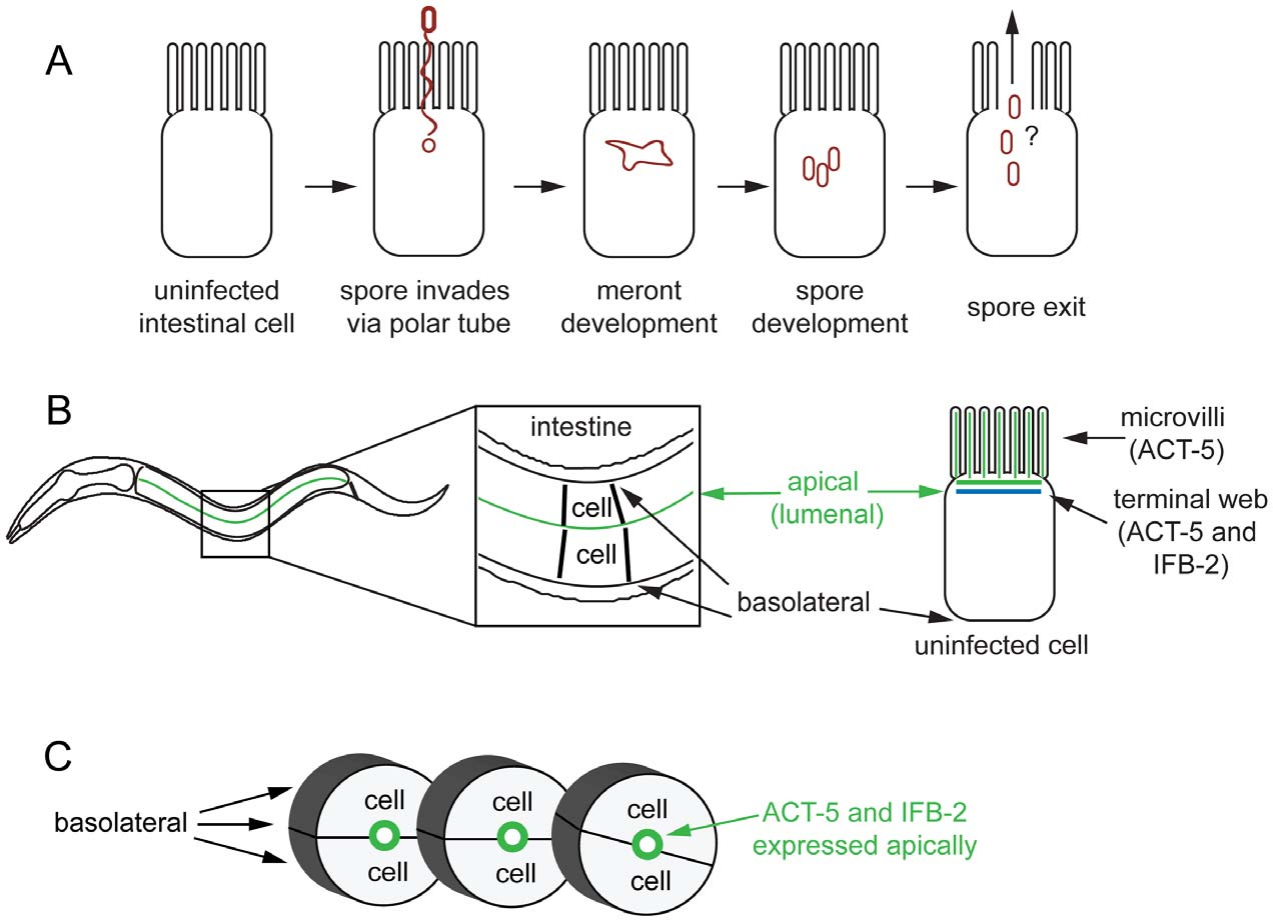
Microsporidian lifecycle and anatomy of *C. elegans* intestinal cells. (A) Generalized microsporidian life cycle inside a host cell. (B) Two dimensional (2D) view of the *C. elegans* intestine. The intestine is formed of polarized epithelial cells, mostly in rings of 2 cells each. On the apical (lumenal) side of these cells are actin-rich microvilli that are anchored into the terminal web, which is composed of actin (ACT-5) and intermediate filaments (IFB-2). (C) Three dimensional (3D) view of three rings of the *C. elegans* intestine (int2, 3, 4), two cells in each ring (other rings not shown for simplicity).

The microsporidia are a diverse group of pathogens that can have very complex life cycles. On a general level, the *N. parisii* life cycle appears similar to that of other microsporidia (Figure 1A) [9], [13]. *N. parisii* infects the *C. elegans* intestine in its transmissible spore form and is transmitted horizontally, likely via a fecal-oral route. After ingestion, microsporidia invade host cells using a specialized infection apparatus called a polar tube, which is coiled inside of the spore and then “fires” to pierce the host cell. The polar tube can inject the nuclei and sporoplasm of the parasite directly into host cells, thereby avoiding extracellular defenses of the host. This injected parasite material develops intracellularly in a replicating cellular structure called a meront. These meronts go through several stages of development and eventually re-differentiate to generate mature spores that somehow must exit the cell and continue the parasite life cycle. *N. parisii* ultimately kills its host, but *C. elegans* can sustain a large spore burden before death. Indeed, live animals can be contagious to their neighbors, indicating that there is a mechanism of exit that does not cause severe damage to the host. Previously, we had observed gaps in the intermediate filaments of the terminal web in infected animals and hypothesized that they may be part of a regulated exit strategy of *N. parisii* [9]. However, we did not know what initiated these gaps, nor how they related to pathogen development and exit.

Here we show that *N. parisii* infection causes dramatic rearrangement of intestinal actin that is part of a novel two-phase, non-lytic exit strategy. The first phase involves restructuring of the terminal web, which is a barrier that pathogens must cross to exit cells. During this phase the subcellular localization of actin is altered, with the apically-restricted actin appearing ectopically on the basolateral side of host cells and forming networks. Subsequently, gaps appear in the terminal web, as assessed by IFB-2, an intermediate filament component of the terminal web. We hypothesize that actin redistribution away from the apical membrane may trigger these gaps in the terminal web, since lowering actin levels causes terminal web gaps in the absence of infection. Soon after the appearance of gaps, the parasite enters the second phase of the exit strategy. During this phase *N. parisii* spores are able to exit the cell. Interestingly, we find that host actin promotes spore exit and that decreasing the levels of actin impairs spore exit. Our analyses of cell integrity indicate that despite very large numbers of spores exiting *C. elegans* intestinal cells, these cells do not lyse. We propose that a novel, two-phase, non-lytic exit strategy allows *N. parisii* to escape from host intestinal cells while minimizing damage to its host, thus increasing parasite transmission.

## Results

### *C. elegans* Actin Ectopically Relocalizes in Intestinal Cells during *N. parisii* Infection

**Figure 2:**
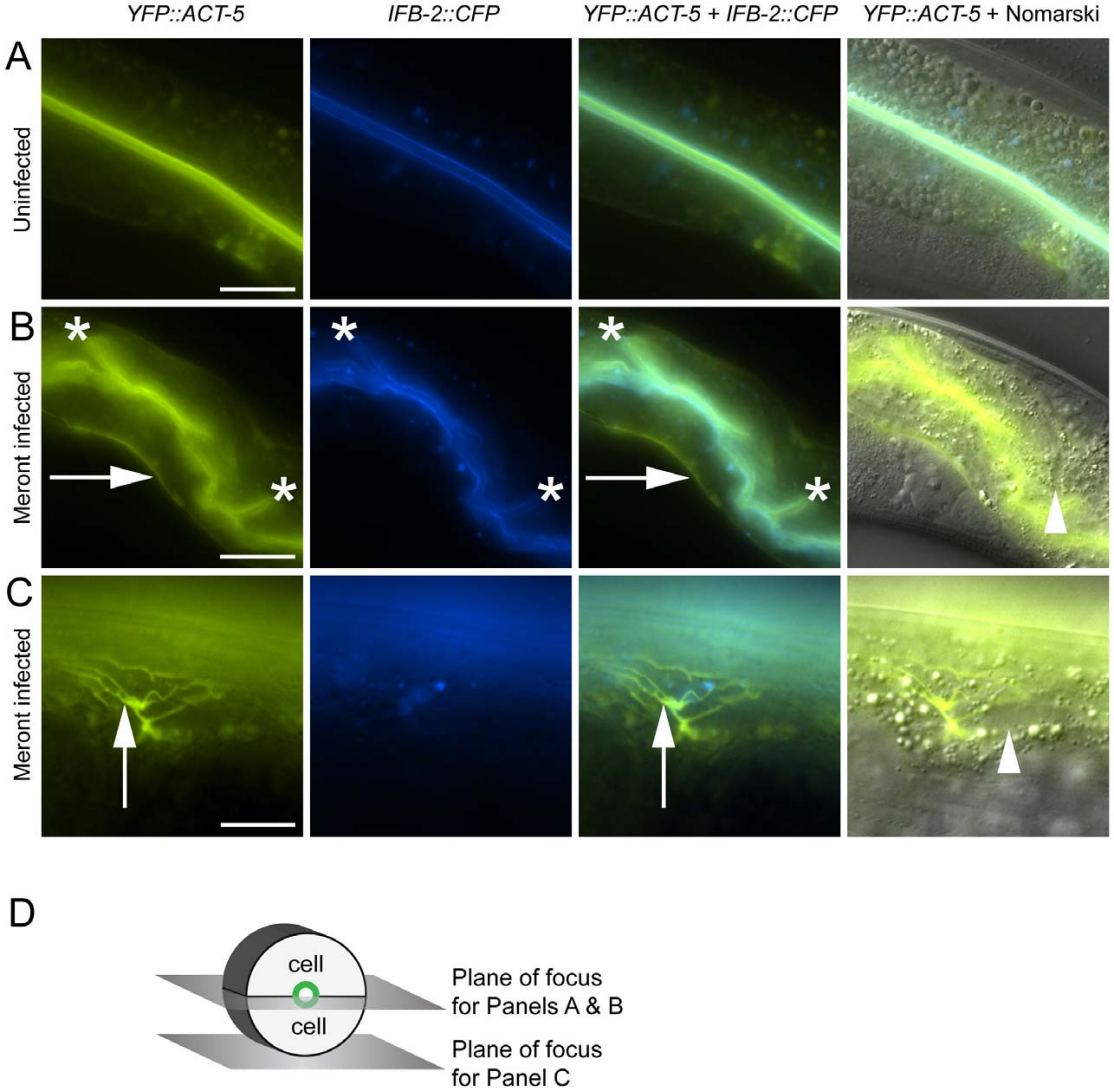
Host intestinal actin relocalizes basolaterally during *N. parisii* meront development. (A) Uninfected animals have apical colocalization of *YFP::ACT-5* and *IFB-2::CFP*. (B) Meront-infected animals exhibit ectopic basolateral localization of *YFP::ACT-5* (indicated by rightward arrows), without concomitant *IFB-2::CFP*. Infected animals often exhibit additional involutions of the lumen as indicated by asterisks, and later during infection exhibit gut distension (not shown). (C) Meront-infected animals exhibit ectopic *YFP::ACT-5* networks (upward-pointing arrows), again in the absence of *IFB-2::CFP*. Meronts are observed as clearings of gut granules, as indicated by arrowheads in Nomarski overlays of (B) and (C). Scale bar for (A, B) is 20 µm, for (C) is 10 µm. (D) Diagram of the 2D planes of focus for (A–C) shown in the context of 3D intestinal cell rings.

*N. parisii* infection proceeds through distinct stages in *C. elegans* intestinal cells, starting with the meront stage when the parasite is actively replicating (Figure 1A). *N. parisii* meronts appear to develop in direct contact with the host cytoplasm, essentially creating “parasite organelles”, which eventually develop into mature spores [9]. Both meronts and spores form inside *C. elegans* intestinal cells, which are polarized epithelial cells that are found in rings of two to four cells that form a tube [6]. Like mammalian intestinal cells, these cells are decorated on the apical (lumenal) side with microvilli that are anchored into a cytoskeletal structure called the terminal web. The *C. elegans* terminal web contains a specialized actin isoform called ACT-5 and an intermediate filament component called IFB-2 (Figure 1B, C). ACT-5 is restricted mostly to microvilli-containing cells and is localized to both the microvilli and the terminal web [23], whereas IFB-2 is restricted to the terminal web [24], [25]. To examine how *N. parisii* infection modifies the intestinal cytoskeleton, we generated a strain that expresses *YFP::ACT-5* as well as *IFB-2::CFP* in the intestine to track both of these cytoskeletal components simultaneously. In uninfected animals, we found that *YFP::ACT-5* colocalized with *IFB-2::CFP* at the apical side of intestinal cells (Figure 2A), consistent with previous reports [24], [25].

We next imaged these cytoskeletal components during infection with *N. parisii*. Because *N. parisii* cannot be propagated outside of host cells, we prepare infectious spores by mechanically disrupting infected animals and purifying the spores away from other material. By adding a preparation of these spores onto plates regularly used to maintain *C. elegans*, the animals become infected by simply ingesting the spores. Once ingested, the spores then presumably fire their polar tubes, injecting *N. parisii* sporoplasm and nuclei into the *C. elegans* intestinal cells. We investigated cytoskeletal protein localization as *N. parisii* infection progressed and found that infection with *N. parisii* caused dramatic changes in ACT-5 localization without concomitant changes in IFB-2. The first relocalization of ACT-5 occurred during *N. parisii* meront development, which is the first stage of parasite development. At this point, we found that ACT-5 localization no longer remained restricted to the apical side of intestinal cells, but appeared ectopically on the basolateral side of cells (Figure 2B). Coinciding with this ectopic basolateral localization that appeared as an unbranched line, we also observed more complex networks of ACT-5 expression (Figure 2C). These networks exhibited branches that varied in number and length, and also appeared basolaterally. The exact molecular nature of these structures is unknown, so we refer to them generally as “actin networks”. They may be part of the same basolateral relocalization phenomenon shown in Figure 2B, but are more easily visualized in the plane of focus shown in Figure 2D because of the orientation of the animal on the slide. In both cases, actin still remained apically localized, although often at lower levels. Videos S1 and S2 show a Z-series of *YFP::ACT-5* localization in the intestine to provide a three-dimensional view of this actin relocalization phenomenon. In general, we did not observe IFB-2 relocalizing with ACT-5 (Figure 2B and 2C), indicating that *N. parisii* infection directs a specific relocalization of actin.

To determine whether these ectopic patterns of ACT-5 localization were general responses to pathogen infection or were specific to *N. parisii* infection, we analyzed *YFP::ACT-5* localization in animals infected with other intestinal pathogens. The Gram-negative bacterial pathogens *Pseudomonas aeruginosa* and *Salmonella enterica* also cause lethal intestinal infections in *C. elegans* [26], [27], [28], [29]. We did not observe ectopic actin basolateral localization or networks in animals infected with *P. aeruginosa* (n = 55 animals), or *S. enterica* (n = 154 animals), indicating that the previously described localization patterns are somewhat specific to infection by the natural intracellular parasite *N. parisii*. We also examined a larger number of uninfected animals and saw only one out of 200 animals that had a single ectopic actin structure without obvious branching, further supporting our finding that this dramatic actin relocalization is specific to *N. parisii* infection.

### Actin Relocalization Precedes Restructuring of Intermediate Filaments in the Terminal Web

**Figure 3:**
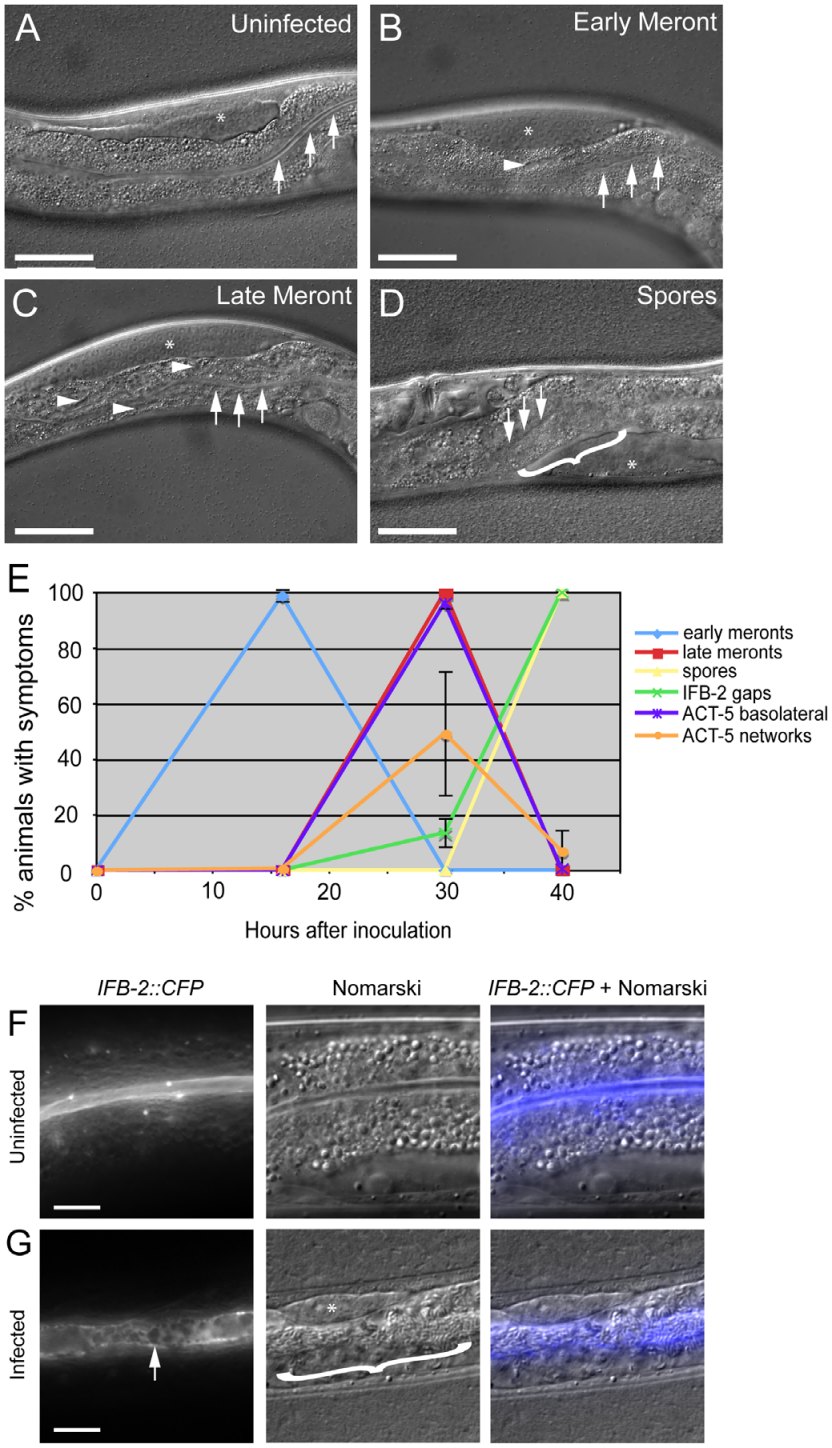
Actin relocalization occurs before terminal web restructuring. (A–D) Stages of *N. parisii* development during infection of *C. elegans* intestine. Three arrows indicate the intestinal lumen and asterisk indicates the germ line. Scale bar is 50 µm. (A) Uninfected animal. (B) Animal infected at early meront stage. Small number of meronts indicated with arrowhead. (C) Animal infected at late meront stage. Meronts are found throughout the intestine; three examples are indicated with arrowheads. (D) Animal infected with spores. Bracket in D indicates region of intestine filled with spores. (E) Kinetics of cytoskeletal restructuring and parasite development in a population of animals infected at time = 0 hours. ACT-5 ectopic localization basolaterally and in branched networks occurs before the appearance of gaps in IFB-2 localization, which occur before and in conjunction with *N. parisii* spore formation. Data shown are the average of three independent experiments with 50 animals scored at each timepoint in each experiment (for a total of at least 150 animals scored at each timepoint). Error bars are SD. (see Table S1 for separate longitudinal infection studies of ACT-5 and IFB-2 in individual animals over time). (F) Uninfected animals exhibit uniform expression of *IFB-2::CFP* in the terminal web on the apical side of intestinal cells. (G) *N. parisii* infection causes gaps in *IFB-2::CFP* expression in the terminal web. Arrow indicates gaps in *IFB-2::CFP,* bracket indicates *N. parisii* spores. Scale bars in (F, G) are 10 µm.

To further characterize this actin relocalization in infected animals, we examined the kinetics of ACT-5 ectopic localization with respect to changes in IFB-2 and to progression of *N. parisii* development. We infected a synchronized population of *YFP::ACT-5;IFB-2::CFP* animals and tracked both *N. parisii* parasite development and changes in these cytoskeletal markers in a population over time (Figure 3). The stages of parasite development are shown in Figure 3A–D. We subdivided the meront stage into “early meront” (Figure 3B), where there was only a small area of gut granule clearing in the intestine, and “late meront” (Figure 3C), where there was more extensive clearing throughout the intestine. Following meront development, spores become visible (Figure 3D). Early meronts appeared by 16 hours post-inoculation (hpi), late meronts appeared by 30 hpi and spores appeared by 40 hpi (Figure 3E). The first obvious change in the cytoskeleton was the ectopic localization of *YFP::ACT-5* (Figure 3E), appearing as a line on the basolateral side of intestinal cells (as shown in Figure 2B). This pattern was observed in all animals of a population during the late meront stage, at 30 hpi. In addition to this linear basolateral relocalization, there was also evidence of ACT-5 networks at this time (Figure 2C and Video S2), continuing at a low level until 40 hpi when all animals contained spores.

We also examined how this ectopic actin localization correlates with restructuring of the terminal web by assessing IFB-2 localization in these same animals. Previously, we had identified gaps in the terminal web of infected animals using transmission electron microscopy (TEM) and gaps in IFB-2 localization using anti-IFB-2 antibodies [9]. Here, we found very similar-appearing gaps in infected animals using CFP-tagged IFB-2 (*IFB-2::CFP)* (compare uninfected animals in Figure 3F to heavily infected animals in Figure 3G). We first observed these gaps in 13% of animals infected at 30 hpi, the late meront stage of infection (Figure 3E). At 40 hpi, when all animals were infected at the spore stage, gaps were found in the terminal web of 100% of animals (Figure 3E). Based on these observations, it appears that actin relocalization occurs before IFB-2 restructuring during infection. We saw a similar pattern of IFB-2 restructuring with respect to parasite development in single *IFB-2::CFP* transgenic animals, indicating that the presence of the *YFP::ACT-5* transgene did not affect *IFB-2::CFP* expression or localization (Figure S1).

The studies described above were performed with a population of animals, which is informative, but does not provide insight into the progression of symptoms within an individual animal. To determine whether the trends we observed in a population (e.g. ACT-5 is relocalized before IFB-2 localization is disrupted) were also true in an individual, we performed longitudinal studies. Individual animals were mounted on slides at varying timepoints for viewing, and then recovered onto plates in between these timepoints to allow them to recover and to allow the infection to progress (Table S1). Consistent with the population studies, these experiments showed that *YFP::ACT-5* basolateral relocalization occurred first, followed by gaps in *IFB-2::CFP* localization.

Our previous studies with IFB-2 antibodies had suggested that terminal web gaps were specific to *N. parisii* infection – we did not observe them in animals infected with other pathogens that cause lethal intestinal infections, such as *P. aeruginosa* and *Staphylococcus aureus* [9]. Using the *IFB-2::CFP* transgene we also analyzed the terminal web in animals infected with the bacterial pathogen *S. enterica*, but did not find evidence that infection caused gap formation in the terminal web (n = 154 animals). To further confirm that terminal web gaps were a *N. parisii*-induced phenotype, we used the *IFB-2::CFP* transgenic animals and performed a thorough inspection of IFB-2 localization in uninfected animals (n = 200 animals). We found that only one animal exhibited a gap, and this was a single, isolated gap. For our analyses of *N. parisii* infected animals, we only scored animals as positive for gap formation if we observed multiple gaps. Thus, similar to the ACT-5 relocalization phenotype, the gaps observed in *IFB-2::CFP* localization appear to be specific to *N. parisii* infection.

### Perturbations in Actin Can Cause Terminal Web Gaps in the Absence of Infection

**Figure 4:**
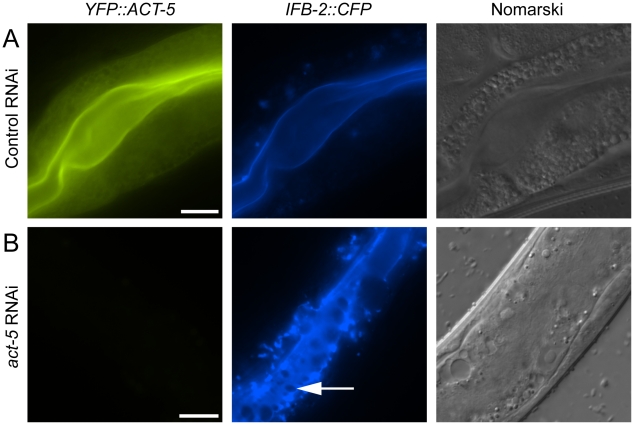
Reducing expression of actin causes terminal web gaps in the absence of infection. (A) Expression of *YFP::ACT-5* and *IFB-2::CFP* in control (L4440 vector alone) RNAi-treated animals. (B) *act-5* RNAi reduces expression of *YFP::ACT-5* and causes gaps in *IFB-2::CFP* expression in the absence of infection, as indicated by arrow. Exposure times were the same between (A) and (B). Scale bar is 20 µm.

Since relocalization of ACT-5 during *N. parisii* infection preceded the appearance of gaps in IFB-2 localization, we hypothesized that reduced levels of ACT-5 in the apical membrane might cause IFB-2 terminal web gaps. To test this hypothesis we reduced ACT-5 expression with feeding RNA interference (RNAi) against *act-5.* We observed a substantial reduction in *YFP::ACT-5* expression in *act-5* RNAi-treated animals compared to control-treated animals, confirming the efficacy of *act-5* RNAi (Figure 4A, B). Interestingly, when we analyzed the terminal web in *act-5* RNAi-treated animals we found gaps in *IFB-2::CFP* that appeared in the absence of infection (Figure 4B). Therefore, we speculate that infection-induced reduction of ACT-5 at the apical membrane may be a triggering event that results in the disruption of the terminal web.

### All Contagious Animals Have Terminal Web Gaps, Which Form during a Discrete Stage of Infection

**Figure 5:**
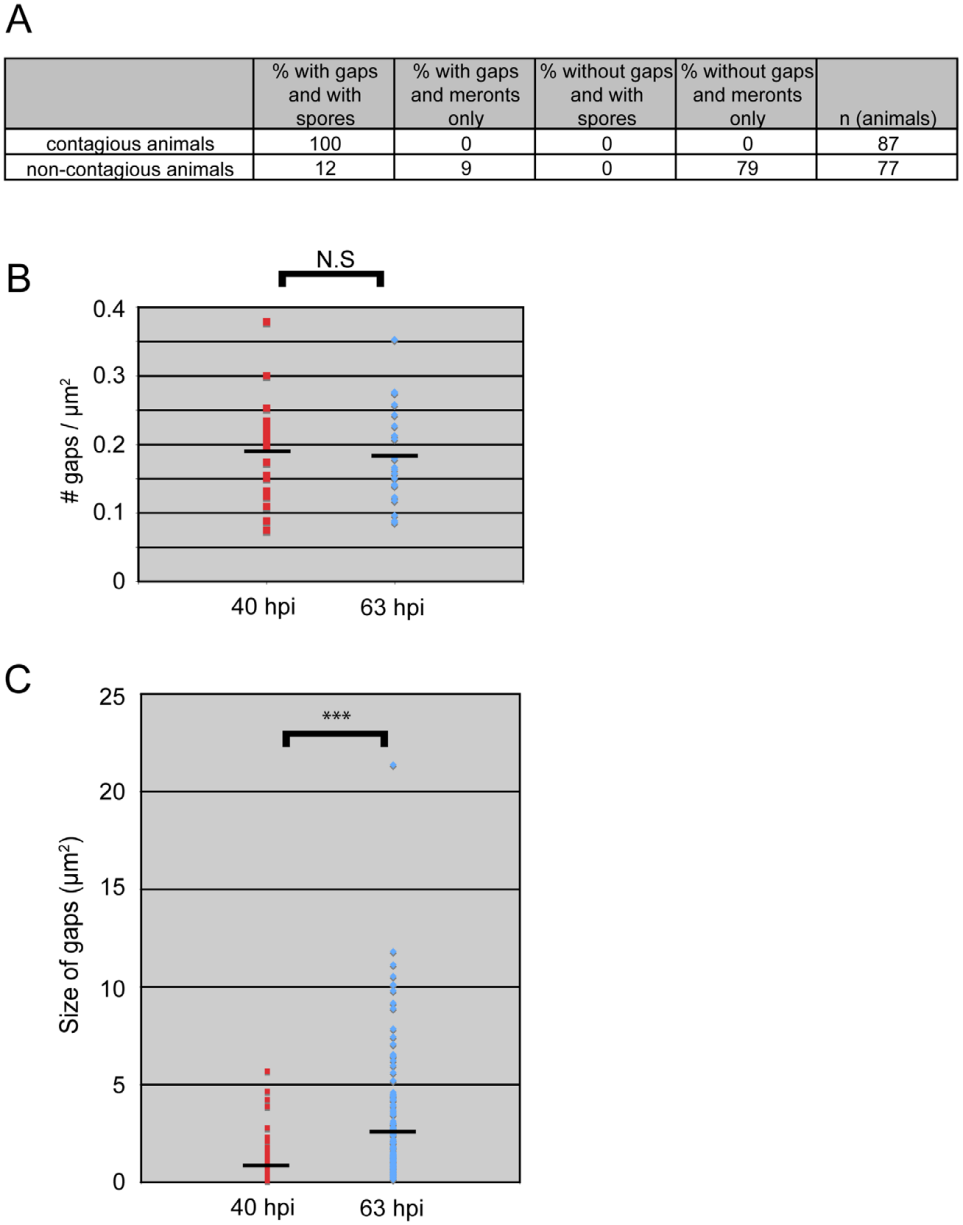
All contagious animals have *IFB-2::CFP* terminal web gaps, which do not increase in number over time but increase in size. (A) Single animal contagiousness assays. Individual “donor” infected *IFB-2::CFP* animals were tested for contagiousness by exposing them to “recipient” uninfected animals. Donors were examined for *IFB-2::CFP* terminal web gaps and scored for stage of infection. 100% of contagious animals exhibited gaps in *IFB-2::CFP* localization and *N. parisii* spores. (B) The number of gaps in *IFB-2::CFP* localization does not change over time. Each datapoint represents one animal (n = 20 animals at each timepoint) where the number of gaps was counted in a defined region of the intestine to calculate the number of gaps/µm^2^. Average number of gaps at 40 hpi is 0.19 gaps/µm^2^ and at 63 hpi is 0.18 gaps/µm^2^ (difference is not significant, p = 0.64, two-tailed *t*-test). (C) The size of gaps in *IFB-2::CFP* localization increases with time. Each datapoint represents the area of one gap, with several gaps quantified per infected animal. At 40 hours post-inoculation (hpi) n = 119 gaps and n = 20 animals; at 63 hpi n = 144 gaps and n = 20 animals. Average gap size at 40 hpi is 0.87 µm^2^ and at 63 hpi is 2.45 µm^2^ (*** difference is significant, p<10^−7^, two-tailed *t*-test). See Materials and Methods for more details on the quantitation method. Data graphed in Figure 5B and C are the combination of two independent experiments: 1) A population study examining two different sets of animals at each timepoint (Table S2), and 2) A longitudinal experiment where we followed single animals at two time points during infection and quantified the gaps in each animal (Table S3). We saw very similar trends within individual animals as we saw within a population of animals in that the number of gaps did not change over time, but gaps increased in size.

Our studies of *N. parisii* parasite development and host cell restructuring suggested that terminal web gap formation is an important stage of the infection cycle. Therefore, we investigated whether terminal web gaps have a functional role by examining how they relate to spore exit. As a functional read-out for spore exit, we tested the contagiousness of individual infected animals: in order for an animal to be contagious, spores must exit intestinal cells, leave the intestinal tract (likely through normal defecation), and then be consumed by a neighboring animal. To measure contagiousness, uninfected “recipient” animals were exposed to individual infected “donor” animals for a defined length of time. Each individual donor animal was then removed and examined for stage of infection and whether it had gaps in *IFB-2::CFP* localization. Several days later, recipient animals were examined to determine whether they had become infected, thus implying that the donor animal had shed spores and was contagious. After testing 164 individual donor animals for contagiousness, we found 87 animals that were contagious and all of these animals had spores, consistent with previous studies. Strikingly, we found that 100% of these contagious animals exhibited terminal web gaps (Figure 5A), consistent with the model that gaps are functionally related to spore exit. Of the animals that were non-contagious, 12% of them exhibited a small number of spores (and gaps). In previous studies we had found that animals infected with only a small number of spores are not always contagious, likely due to a low amount of spores being shed before the donors were removed from the plate [9]. There were also 9% of non-contagious animals that exhibited gaps but only meronts (no spores), consistent with population studies that indicated gaps appear slightly before spore formation (Figure 3E). The remaining 79% of animals that were non-contagious exhibited no gaps and only meronts (no spores). Since all contagious animals exhibit terminal web gaps, these data support the model that terminal web gaps may be a necessary part of spore exit from the intestinal cells.

We next sought to determine whether new gaps are continually being made during the course of infection, or whether gap formation is a discrete event that occurs only once during infection. In general, gaps first appeared in the region of the intestine exhibiting the heaviest *N. parisii* infection and gaps were always present in the region of intestine that had spores (Table S1 and data not shown). To quantify the number of gaps over time we chose a plane of focus with the largest number of gaps from *N. parisii* spore-infected animals and counted the number of gaps at 40 hpi, when they first are visible in all animals, and again at 63 hpi. With this analysis, we found that the number of gaps per unit area remained similar as the infection proceeded, suggesting that new gaps are not made after their initial formation at 40 hpi (Figure 5B). Next, we quantified the size of gaps at 40 hpi and 63 hpi (see Figure legend and Experimental Procedures for more detail on quantification). With this analysis, we found that gaps became larger as the infection proceeded, suggesting that once a gap is made it becomes larger over time (Figure 5C). This effect may be due to the lumenal distention that is observed as infection progresses, or it may be due to a more active process. In any case, these data indicate that the formation of gaps appears to be a discrete, regulated event that is orchestrated by the parasite at a particular stage in infection.

### Reducing Levels of Host Actin Impairs *N. parisii* Spore Exit

**Figure 6:**
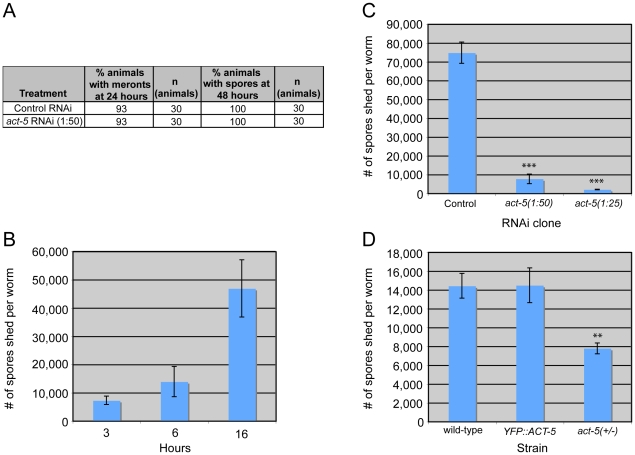
Host actin is required for spore shedding. (A) Infection kinetics in RNAi-treated animals. *N. parisii* development proceeds similarly in control (L4440) and *act-5* RNAi-treated animals. Results are representative of three independent experiments, with at least 25 animals in each condition of each experiment. (B) Spore shedding assay shows increasing number of spores shed by animals over time. Error bars are SD to show variance in assay. (C) *act-5* RNAi inhibits spore shedding, compared to control (L4440) treated animals. 1∶25 diluted *act-5* RNAi inhibits shedding more than 1∶50 diluted *act-5* RNAi. Animals shed spores for 16 hours in this assay. (D) *YFP::ACT-5* animals shed the same number of spores as wild-type animals, while *act-5(ok1397)/mT1[dpy-10(e128)]* heterozygote mutants shed 50% fewer spores than wild-type animals. Animals shed for 13 hours in this assay. (C-D) Number of spores shed per worm is the average of three replicates in a single experiment (with 50 animals tested in each replicate), error bars are SEM. Results are representative of three independent experiments. (*** indicates p<0.0005, ** indicates p<0.002, two-tailed *t*-test).

The extensive relocalization of ACT-5 during infection and the induction of gaps in IFB-2 localization upon RNAi against *act-5* led us to investigate the functional role of ACT-5 during infection. First, we assessed whether reducing levels of actin would affect the development of *N. parisii* infection inside intestinal cells. We measured the progression of *N. parisii* infection in animals fed with bacteria expressing dsRNA against *act-5*. Because feeding undiluted *act-5* dsRNA-expressing bacteria can slow the growth of animals, we titrated back the dose of RNAi to allow animals to develop more normally. Diluting *act-5* RNAi bacteria 1:25 and 1:50 with control bacteria allowed worms to develop relatively normally, but still caused reduced *act-5* levels (as assessed by imaging *YFP::ACT-5,* Figure S2). We found that meronts and spores appeared in *act-5* RNAi-treated animals at a similar rate to control-treated animals (Figure 6A). Therefore, despite the changes induced in actin localization upon infection, reduction of *act-5* expression had no obvious effect on progression of *N. parisii* infection or formation of spores within the host cells.

Since reducing actin in the absence of infection caused gaps in the terminal web, which could be a barrier for exit, we speculated that reducing actin might cause animals to shed more spores. Thus, we sought to measure spore exit in *act-5*-defective animals. Previously, we used single animal contagiousness assays to measure spore exit (Figure 5A). However, these assays are labor-intensive and categorical (animals are binned as either contagious or non-contagious). Therefore, we developed a more quantitative “spore shedding” assay to measure the number of spores shed by a population of animals. Fifty infected animals were placed in liquid media containing *E. coli* (the normal food source of *C. elegans*) and allowed to shed spores into the media. After defined periods of time, the number of spores shed was quantified. Animals appeared to shed spores throughout the assay, as we found increasing numbers of spores were shed with increasing amounts of time (Figure 6B). Under these assay conditions we found that a single infected worm could produce thousands of spores per hour. However, the number of spores shed per animal had significant animal-to-animal variability (data not shown), so we performed assays with populations of animals. Using this assay, we tested whether reducing ACT-5 levels would increase *N. parisii* spore shedding. Surprisingly, we found that spore shedding was almost completely blocked in 1∶25 *act-5* RNAi-treated animals and was substantially blocked in 1∶50 *act-5* RNAi-treated animals compared to control-treated (L4440) animals (Figure 6C). These findings suggest that reduction of ACT-5 impairs spore exit, in contrast to our original hypothesis. Altogether, our experiments indicate that proper levels of host actin are not important for *N. parisii* development inside cells, but are critical for promoting spore exit from those cells.

To further test the hypothesis that actin promotes spore exit we examined two other *C. elegans* strains that have differing levels of *act-5* expression. First, we examined the *YFP::ACT-5* transgene, which likely overexpresses actin. Our experiments indicated that *YFP::ACT-5* had no effect on the levels of spore shedding in our assays compared to wild-type animals (Figure 6D). Next we examined the *act-5(ok1397)* deletion mutant, which is likely a null mutation, as it deletes half of the 5′ translated region of *act-5* as well as the upstream untranslated region. Because the *act-5(ok1397)* mutation is lethal when homozygous, we examined spore shedding in *act-5(ok1397)/+* heterozygote mutants, which develop normally. We infected these animals with *N. parisii* and found that spore development proceeded at a similar pace as in wild-type animals (data not shown). Interestingly, in *act-5* heterozygote mutants where *act-5* expression should be reduced to half of wild-type levels, we found that spore shedding was approximately half of wild-type levels (Figure 6D), consistent with our hypothesis that actin promotes exit. This result suggests that there is a dose-dependent effect of actin on spore shedding, and that proper levels of actin are critical for efficient *N. parisii* spore exit from host cells.

### Spores Exit Apically from Intestinal Cells and Appear to be Free of *C. elegans* Membrane

**Figure 7:**
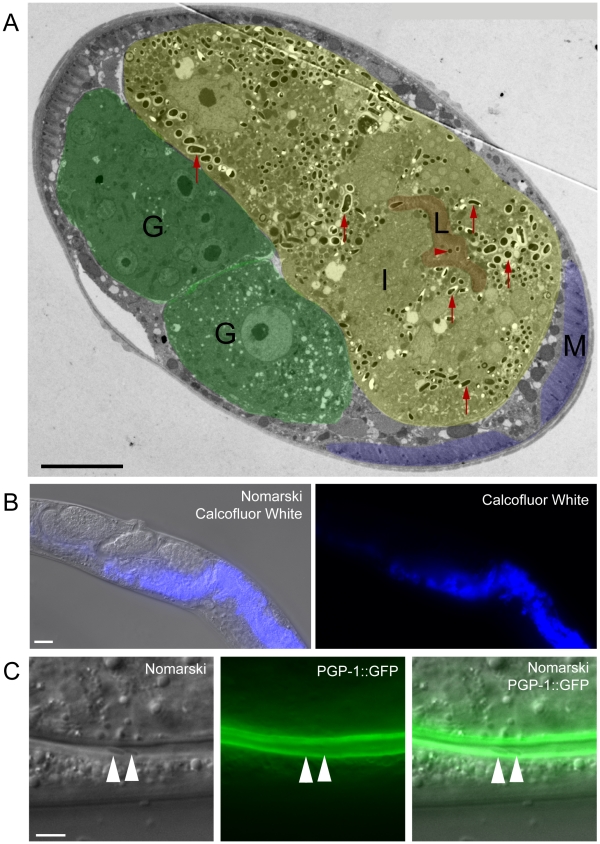
*N. parisii* spores exit apically out of intestinal cells and are not labeled with a *C. elegans* membrane marker. (A) TEM indicating *N. parisii* spores inside intestinal cells and the intestinal lumen. Intestinal cells are false colored yellow (labeled I), the intestinal lumen and microvilli are false colored red (labeled L), the gonad is false colored green (labeled G) and body wall muscle is false colored purple (labeled M). Examples of intracellular spores in the intestinal cells are indicated with red upward arrows and an example of a lumenal extracellular spore is indicated with red rightward arrowhead. Spores were only observed inside intestinal cells and in the intestinal lumen, suggesting that they only exit apically from intestinal cells (not basolaterally). Scale bar is 10 µm. (B) *N. parisii* infected animal fixed and stained with Calcofluor White to label *N. parisii* spores inside intestinal cells. Scale bar is 20 µm. (C) *N. parisii* spores in the intestinal lumen of *PGP-1::GFP* animals are free of GFP, suggesting that they did not take *C. elegans* membrane upon exit into the lumen. Two spores in lumen are indicated with arrowheads. *PGP-1::GFP* labels the apical (lumenal) side of two intestinal cells. Scale bar is 5 µm.

The spore shedding and contagiousness assays described above measure the spores that have left the animal, presumably through defecation. Before these spores are shed through defecation, they must first exit the intestinal cells into the lumen. Our previous studies indicated that spores are only found in intestinal cells, and spores are therefore unlikely to exit out of cells basolaterally to enter the rest of the animal (e.g. into the gonad). To more closely examine that spores only exit apically as opposed to basolaterally, we performed transmission electron microscopy (TEM) to analyze the location of spores in the whole animal. The only tissue in which we conclusively found spores was the intestine (Figure 7A); we did not see evidence of spores in any other tissue (e.g. hypodermis, muscle or gonad). In order to further examine spore location throughout the animal, we also fixed animals and stained with Calcofluor White, a dye that binds to the chitin found in *N. parisii* spores. Again, we only found evidence of *N. parisii* spores in the intestine, not in other regions of the animal (Figure 7B).

Because *N. parisii* spores have to cross the *C. elegans* apical plasma membrane of intestinal cells to exit into the lumen, we investigated whether spores in the lumen may have acquired *C. elegans* plasma membrane upon exit. To address this question we infected transgenic animals expressing *PGP-1::GFP*, which is a GFP-tagged ATP binding-cassette (ABC) transporter that localizes to the apical side of intestinal cells [30]. After infecting worms, we transferred them repeatedly away from spores onto fresh plates, so that any spores in the lumen were likely the result of exit and not the result of consumption (see Materials and Methods for details). We did not find any lumenal *N. parisii* spores that were labeled with GFP (Figure 7C, n = 50 spores examined), suggesting that spores do not take *C. elegans* membrane with them when they exit into the lumen.

### *N. parisii* Spores Can Exit Intestinal Cells without Causing Lysis

**Figure 8:**
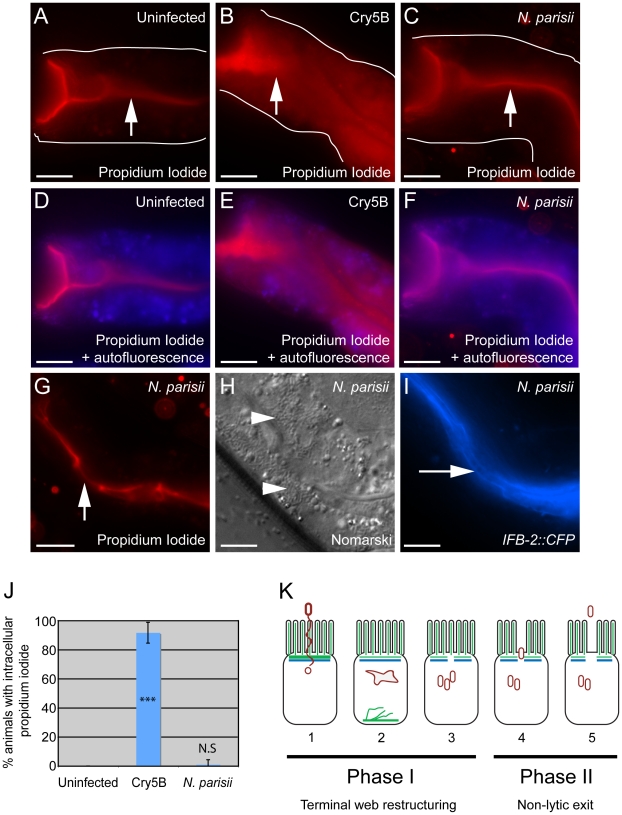
Animals infected with *N. parisii* spores do not take up the extracellular dye propidium iodide. Propidium iodide staining in uninfected animal (A, D), animal treated with Cry5B pore-forming toxin (B, E), and animal infected with *N. parisii* spores (C, F). Intestinal cells encompass the entire space between the lumen (indicated with upward arrows) and the basolateral side of intestinal cells (indicated with outlines). Outlines were drawn based on Nomarski images, which are shown in Figure S3 in order to better illustrate intestinal cell morphology. The intracellular region is also indicated with an overlay the autofluorescence in the blue channel to indicate intracellular regions of intestinal cells (D–F). Note the extensive propidium iodide staining filling up the intracellular region in Panels B and E. (G) Propidium iodide staining in an *N. parisii* infected animal with upward arrow indicating lumen. (H) Nomarski image of animal shown in G, arrowheads indicate *N. parisii* spores; (I) *IFB-2::CFP* of animal shown in G and H, rightward-pointing arrow indicates gaps in the terminal web; Scale bar is 20 µm in A–I. (J) Percentage of animals with intracellular propidium iodide shown as the average of 4 independent experiments, n = 100 animals total for each condition, error bars are SD. The difference between uninfected and Cry5B treated animals is significant, *** indicates p<10^−6^ with a two-tailed *t*-test. The difference between uninfected and *N. parisii*-infected animals is not significant (p = 0.36). (K) Model for actin-based, non-lytic *N. parisii* spore exit from *C. elegans* intestinal cells has two phases in five steps: 1) *N. parisii* spores invade *C. elegans* intestinal cells, likely with polar tube. 2) *N. parisii* meronts develop and ACT-5 ectopically localizes to the basolateral side of cell and in branched networks. This may lead to less expression on the apical side. 3) Gaps appear in the terminal web, then *N. parisii* spores form. 4) Spores exit the cells, causing worms to be contagious. 5) Exit is non-lytic, because the extracellular dye propidium iodide is unable to enter cells.

It is clear that *N. parisii* spores are exiting while animals are still alive, as live animals are contagious to other animals ([9] and Figure 5A) and animals are alive at the end of spore shedding assays (Figure 6). However, it is unclear whether this process of spore exit causes tissue damage that leads to cell lysis. To address this question, we next examined whether *N. parisii* spore exit and/or terminal web restructuring cause disruption of cellular integrity of the intestinal cells. To assay cellular integrity, we used the cell-impermeable dye propidium iodide (PI), which can be fed to *C. elegans.* When intestinal cells are intact, PI does not enter cells after short incubation periods, but instead remains restricted to the intestinal lumen. As a positive control for PI entry into cells that have lost integrity, we used animals that were exposed to the *Bacillus thuringiensis* pore-forming toxin Cry5B, which creates nanometer-sized holes in the plasma membrane. A recent study demonstrated that Cry5B treatment of *C. elegans* creates pores in intestinal cells that allow PI to enter intracellularly [31]. Consistent with these studies, we found that animals fed *E. coli* that expresses Cry5B had intracellular PI staining whereas animals fed normal *E. coli* did not (compare Figure 8A and B). Strikingly, animals infected with *N. parisii* (also fed on *E. coli*) did not exhibit intracellular PI, indicating that cellular integrity was not compromised (Figure 8C and G). These animals were infected with *N. parisii* spores (Figure 8H), and exhibited gaps in *IFB-2::CFP* localization (Figure 8I). In order to quantify this effect, we scored the percentage of animals with intracellular PI in 100 animals of each condition, only choosing animals that were infected throughout the intestine with spores for the *N. parisii* infection condition. From single-animal contagiousness assays with animals at a similar stage of infection (Figure 5A), we estimate that 80-90% of these animals were contagious. In these studies we found that almost all Cry5B-treated animals exhibited intracellular PI, whereas virtually none of the *N. parisii*-infected animals did (Figure 8J). Thus, while *N. parisii* infection causes gaps in the terminal web and results in the passage of 1 µm-wide *N. parisii* spores (much larger than Cry5B-induced pores) across the membrane in order to exit into the lumen, these events do not appear to cause lysis of *C. elegans* intestinal cells. These data support the hypothesis that cytoskeletal rearrangements and spore exit are highly regulated processes that maintain the integrity of the host cell during infection.

## Discussion

Intracellular pathogens have privileged access to host cell components that they can exploit in order to survive, replicate, and then exit to infect new hosts. The studies presented here provide insight into parasite-directed cellular reorganization that occurs *in vivo* in order to exit cells but minimize damage to the host. We find that the intestinal parasite *N. parisii* redirects host actin in order to non-lytically exit from intestinal cells and propose a two-phase model for this exit, with terminal web restructuring occurring during Phase I and non-lytic exit occurring during Phase II (Figure 8K). Phase I takes place after invasion (step 1), when *N. parisii* is developing as a metabolically active meront, a stage that essentially forms a new organelle within the host cell. At this time, the intestinal-specific actin isoform ACT-5 ectopically relocalizes to the basolateral side of intestinal cells, often forming network-like structures (step 2). Soon after this relocalization of ACT-5, gaps appear in localization of IFB-2, an intermediate filament component of the terminal web (step 3). We speculate that relocalization of ACT-5 may trigger these gaps, since reducing the level of ACT-5 expression causes gaps in the terminal web in the absence of infection. We show that all contagious animals exhibit gaps in localization of IFB-2, and that formation of gaps occurs during a discrete stage of the infection cycle, suggesting that gap formation is a highly regulated process. Phase II of the exit strategy begins after *N. parisii* develops into spores and these spores exit out of cells into the lumenal space (step 4). Surprisingly, we found that reducing levels of ACT-5 impairs spore shedding, indicating that host actin is required for proper exit of *N. parisii* spores at this stage, even though the infection and parasite development can proceed normally within the cell. Interestingly, *N. parisii* spore exit is non-lytic, since extracellular dyes continue to be excluded from the intracellular space of intestinal cells (step 5). This finding is striking, as a single infected animal can shed thousands of spores (Figure 6).

Our functional analyses of host intestinal actin during *N. parisii* infection indicate that there are two distinct roles for actin, with opposing effects in each of the two phases of exit. As described above, it appears that host actin impairs exit during Phase I, since a reduction of actin leads to gaps in the terminal web, which may present a barrier to exit. However, we found that a reduction of actin levels led to an overall reduction in spore exit. Thus, one explanation is that host actin impairs spore exit during Phase I, but promotes spore exit during Phase II. A caveat to this interpretation is that the function of terminal web gaps (and thus the role of actin in Phase I) is unclear since we do not yet have a method to block gap formation during infection: it is possible that gaps serve a purpose other than facilitating exit. Our proposed role of actin in Phase II to promote exit also requires further investigation. One possibility is that actin provides motor force to drive spores out of the cell. The nature of this actin polymerization and its relocalization at the proper time may be the key to orchestrating the complex events that enable large numbers of microsporidian spores to egress out of *C. elegans* intestinal cells in a manner that minimizes damage to its host.

Understanding how *N. parisii* is able to exit the host cell necessitates understanding what barriers it must cross and the mechanism it employs to cross them. In Phase I of the *N. parisii* exit strategy, the terminal web is restructured. Our studies indicate that terminal web gaps appear right before spore formation (Figures 3E and 5A), implying that gaps are not caused by the mechanical process of spore exit, but rather by a regulated signaling event that is precisely timed to precede spore formation and the need to exit. The terminal web is a conserved structure found in microvilli-containing cells from *C. elegans* to humans, but surprisingly little is known about its assembly and dynamics [32], [33], [34], [35]. The terminal web presumably represents a barrier that host vesicles must traffic through during normal endocytosis and exocytosis events. Do intracellular vesicles need to dissolve the terminal web in order to cross this barrier? Electron microscopy studies indicate there are tiny gaps in the *C. elegans* terminal web that are too small to allow for vesicle passage, but are associated with vesicular elements and could represent a system of regulated passage [36]. Perhaps *N. parisii* is exploiting this vesicle passage system to create gaps in the terminal web, but in a way that does not result in resealing of these gaps. It will be interesting to perform live imaging of the kinetics of these structures in uninfected animals to examine what is the basal level of movement and restructuring of this conserved structure in intestinal cells. It will also be interesting to further examine the mechanism by which *N. parisii* spores cross the plasma membrane, as our studies indicate that they do not acquire *C. elegans* membrane as they exit into the lumenal space of the intestine (Figure 7C).

In spite of the terminal web restructuring described above, and the passage of 1 µm-wide spores out of cells into the digestive tract, cellular integrity assays indicate that actin-based exit of *N. parisii* spores does not cause lysis of *C. elegans* intestinal cells (Figure 8). This finding is intriguing, since bacterial pathogens such as *Listeria* and *Mycobacterium* also use actin-based, non-lytic forms of exit, suggesting a common evolutionary strategy for exit between eukaryotic and prokaryotic pathogens. Several bacterial virulence factors have been identified that direct these actin-based processes within host cells [4], [37], but comparatively little has been explored about eukaryotic intracellular pathogens and the factors they use to exploit host cells. And almost nothing is known about virulence factors in the Microsporidia phylum. One of the few characterized examples of microsporidian virulence factors is a class of ATP transporters that can “steal” ATP from the host cell [14], [38]. These transporters are expressed on the plasma membrane of the parasite while it is living inside the host cell and act to import ATP directly into the parasite. In addition to this method of host exploitation, it is likely that microsporidia also secrete factors from the meront into the host cytoplasm to facilitate other kinds of nutrient acquisition, as well as to direct the cytoskeletal changes we have observed. The *N. parisii* genome has recently been sequenced (E.R.T. and the Broad Institute, as part of the Microsporidian Genomes Consortium, unpublished data, http://www.broadinstitute.org/files/shared/genomebio/Microsporidia_wp.pdf) and may provide clues into which parasite components are secreted into the host cell to direct this restructuring.

We have isolated microsporidia-infected nematodes from a wide variety of geographical locations, including multiple regions in France (the *N. parisii* strain used in this study was isolated near Paris), Portugal, India, Colombia and Cape Verde ([9] and Marie-Anne Félix, personal communication). Thus, *Caenorhabditis* nematodes have likely co-evolved with *Nematocida* and other microsporidia species. Over time, co-evolution of host/parasite pairs is thought to lead to a reduction in virulence [39], [40]. This pressure is likely to be especially great in obligate intracellular parasites such as microsporidia, which cannot grow in the absence of host cells. Microsporidian species that infect fish appear to have adopted a strategy of minimal virulence in order to maximize parasite production: they often form “spore factories” called xenomas in the fish nervous system that can produce large numbers of spores without substantial impact on the health of the fish [20]. Perhaps the non-lytic exit of *N. parisii* is part of a strategy similar to the xenomas found in fish that serves to maximize spore production and transmission, but minimize virulence.

*N. parisii* likely has an intimate relationship with the *C. elegans* intestinal cell during the meront stage, since it essentially creates a parasite organelle that develops in direct contact with the host cytoplasm [9]. The human-infecting microsporidian pathogen *Enterocytozoon bieneusi* also develops in direct contact with the host cytoplasm during the meront stage [41]. *E. bieneusi* infection in humans is restricted to intestinal cells, and is the most common cause of microsporidian disease in humans, being responsible for lethal diarrhea in AIDS patients [18]. Effective treatments are lacking for *E. bieneusi* infection [15], [42] and it has not been possible to propagate *E. bieneusi* in tissue culture cells, perhaps because these cells lack some aspect of differentiated intestinal cells that is needed for the infection cycle. While *E. bieneusi* and *N. parisii* are in distinct clades of the microsporidia, their similar developmental life cycle in the cytoplasm of intestinal cells may involve similar pathogenic strategies. The *C. elegans*/*N. parisii* host/parasite system may thus provide insights into the mechanisms employed by medically relevant but intractable microsporidian species such as *E. bieneusi* to further understand how they cause disease and potentially how to treat the infections they cause.

## Materials and Methods

### Strains

*C. elegans* were maintained on NGM plates seeded with OP50-1, as described [43]. We used N2 wild-type animals. BJ49 *kcIs6[IFB-2::CFP]* was kindly provided by Olaf Bossinger. JM125 caIs*[ges-1p::YFP::ACT-5]* was kindly provided by James McGhee [24]. ERT38 caIs *[YFP::ACT-5;IFB-2::CFP]* was made by crossing BJ49 and JM125 strains. A separately made *YFP::ACT-5* strain, IN4000 dtIs2298, was kindly provided by James Waddle and used for Videos S1 and S2. VC971 *+/mT1 II; act-5(ok1397)/mT1[dpy-10(e128)] III* was provided by the *C. elegans* Reverse Genetics Core Facility at UBC via the *Caenorhabditis* Genetics Center (CGC). The presence of the *act-5(ok1397)* deletion was confirmed by PCR genotyping. GK288 *GFP::PGP-1* and *E. coli* (OP50)-Cry5B strains were kindly provided by Ferdinand Los and Raffi Aroian. *N. parisii* strain ERTm1 (isolated originally from Franconville, France, near Paris) was used for all microsporidia infection experiments.

### *N. parisii* Spore Preps

*N. parisii* infected animals were disrupted with silicon beads as described [9]. This lysate was then filtered through a 5 µm filter (Millipore) attached to a 10 ml syringe, to eliminate undisrupted *C. elegans* eggs, larvae and other debris. The filtrate containing *N. parisii* spores was frozen at −80°C and then thawed right before use. *N. parisii* spores were quantitated by staining with Calcofluor White (Fluka) and counting with a hemocytometer (Cell-Vu).

### Infection Assays and Microscopy

In general, synchronized L1 larvae were grown for 24 hours on 10 cm NGM plates seeded with OP50 at 20°C until approximately L3 stage, when they were infected with 3×10^7^ *N. parisii* spores and then incubated at 25°C. Symptoms of infection were tracked by mounting approximately 50 animals on agarose pads and then viewing animals with Nomarski optics and/or fluorescence on a Zeiss AxioImager at 630X magnification. Images were captured with Axiovision software. *P. aeruginosa* PA14 infection assays and *S. enterica* SL1344 infection assays were performed as described [44], except that *S. enterica* infection assays included a pre-treatment with *bec-1* RNAi to knock down the autophagy pathway, as described [29]. Transmission electron microscopy was performed as described [9]. Calcofluor White staining on infected animals was performed by fixing and staining with a 1∶1 mix of 1M NaOH and Calcofluor White. In order to examine spores that had newly exited into the intestinal lumen (as shown in Figure 7C), animals were repeatedly washed to reduce exposure to external spores they might ingest. Specifically, at 24 hpi (prior to spore formation) animals were washed four times in M9, added to fresh NGM plates seeded with OP50, and incubated at 25°C until 40 hpi when this process was repeated. To reduce the probability of animals consuming spores shed by their neighbors, worms were replated at a low density of approximately 45 worms per 6 cm plate. Lumen-localized spores were then imaged in animals at 42 hpi.

### Contagiousness Assays

Donor BJ49 *[IFB-2::CFP]* animals were generated for contagiousness assays as follows. Synchronized L1 larvae were grown on 6 cm NGM plates seeded with OP50 for 24 hours at 20°C, then infected with 1.5×10^5^, 4.7×10^5^ or 9.3×10^5^ spores diluted in 500 µl of M9 and placed at 20°C or 25°C for 20 hours in order to generate animals at a variety of infection stages. These animals were picked to new NGM/OP50 plates without spores and incubated for 4 hours, washed off the plate with M9, rinsed three times to remove spores attached to the cuticle and then added to a new plate. Next, these infected donor animals were individually placed onto 6 cm NGM plates seeded with OP50 as well as 200 L1 recipient animals. The donor and recipient animals were co-incubated for 16 hours and the donor animal was then removed to assess infection level by mounting the animal on a slide and scoring for meronts or spores using Nomarski optics, as well as scored for gaps using fluorescence microscopy, at 630X. Recipient animals were scored for infection similarly 7 days later. If no infection was observed, plates were kept and then scored again for infection 3 days later.

### Characterization of Gaps in *IFB-2::CFP* Localization

Animals at 40 and 63 hours post-inoculation (hpi) were analyzed. A 10 µm length of the intestine was chosen such that the wall of the intestine was in a single plane of view. The width of this 10 µm long section was measured and the total number of gaps in *IFB-2::CFP* localization was counted to calculate the number of gaps per µm^2^. This area was then divided into 4 quadrants. In a single, randomly chosen quadrant, the area of each individual gap within the quadrant was quantified using the measure tool in AxioImager in order to calculate the average size of gaps. Statistical comparisons of data at 40 hpi and 63 hpi were done with a two-tailed *t*-test in Excel.

### Longitudinal Infection Assays

ERT38 *[IFB-2::CFP; YFP::ACT-5]* synchronized L1 animals were plated on NGM OP50 plates and infected with 3.6×10^6^ spores 24 hours later. In order to perform longitudinal analysis of infection symptoms in the same animal over time, animals were anesthetized with levamisole and mounted onto agarose slides for scoring of symptoms, and then recovered onto NGM/OP50 plates until the next timepoint.

### RNA Interference Assays

Feeding RNAi was performed as described. Briefly, RNAi bacterial clones were streaked onto LB agar plates with ampicillin and tetracycline. A single colony was inoculated into LB with ampicillin, grown overnight and then seeded on RNAi plates (essentially nematode growth media with IPTG and ampicillin – usually 6 cm plates). Plates were incubated for at least one day before being seeded with a synchronized population of L1 *C. elegans*. *act-5* RNAi was either used undiluted, or diluted 1∶25 or 1∶50 with L4440 (vector alone) control RNAi bacteria. We performed *act-5* RNAi experiments either with a clone from the Ahringer RNAi library or a clone that was a gift from James Waddle [23]. RNAi clones were confirmed by sequencing. Similar results were obtained with both clones, but in general, the Ahringer clone caused more severe phenotypes.

### Spore-Shedding Assays

Animals were infected with 4.5×10^5^ or 9.3×10^5^ *N. parisii* spores on NGM plates seeded with *E. coli* OP50 and placed at 25°C. At 24 hpi, animals were washed with M9 and transferred to new NGM OP50 plates (except when performing RNAi). Then at 48 hpi animals were washed several times and then transferred to a fresh NGM/OP50 plate to remove external spores. Next, 50 animals were picked off of this plate into a microfuge tube with M9 and concentrated OP50, and rotated on a nutator for 16 hours. The microfuge tube was removed from the nutator and incubated without shaking for 10 minutes to allow the *C. elegans*, but not the spores, to pellet to the bottom of the tube. Then the supernatant was transferred to a new microfuge tube and the spores were pelleted by spinning at high speed in a microfuge. The supernatant was removed after this spin and the spores in the pellet were stained with Calcofluor White and quantified with a hemocytometer. The absolute number of spores shed per worm varied from experiment to experiment. The viability of animals was confirmed at the end of the assays and assays were performed in triplicate for each experiment. Statistical comparisons of data were done with a two-tailed *t*-test in Excel.

### Cellular Integrity Assays

Assays for integrity of intestinal cells was performed with a propidium iodide assay, as described [31]. Briefly, *YFP::ACT-5; IFB-2::CFP* animals were infected with 9.3×10^5^ spores on an OP50-seeded 6cm NGM plate. Approximately 40 hours later, animals were washed off the plate and put in a solution of 5-HT at 5 mg/ml for 15 minutes in order to force feed dye into animals – Cry5B treatment causes animals to cease feeding. Animals were then stained in a 20 µg/ml solution of propidium iodide for 30 minutes, washed twice with M9 to remove background excess dye, and imaged using fluorescence microscopy at 630X. We scored only animals that were at the “full spore” stage and therefore were very likely contagious and actively shedding spores. Negative control animals were treated similarly except they were not infected with spores. Positive control Cry5B-treated animals were plated as synchronized L1 larvae on NGM OP50 plates until they reached the L4 stage. They were then washed onto NGM/Ampicillin/IPTG plates seeded with OP50-Cry5B and incubated at 25°C for 30 minutes. They were then washed off the plates and treated as detailed above. In order to quantify the percentage of animals exhibiting propidium iodide staining intracellularly, we imaged animals with a defined exposure time and then measured the maximum fluorescence intensity in the lumen and intracellularly using ImageJ software from the NIH (http://rsb.info.nih.gov/ij/). If the maximum fluorescence intensity was greater intracellularly than in the lumen, the animal was binned as exhibiting intracellular propidium iodide. Statistical comparisons of data were done with a two-tailed *t*-test in Excel.

### Accession Numbers

Accession numbers for the genes and gene products mentioned in this paper are given for Wormbase, a publically available database that can be accessed at http://www.wormbase.org.

These accession numbers are: *act-5 (T25C8.2), ifb-2 (F10C1.7), bec-1 (T19E7.3), pgp-1 (K08E7.9).*

## Supporting Information

Figure S1 **Terminal web restructuring in** ***IFB-2::CFP*** **single transgenic animals.** Kinetics of terminal web restructuring and parasite development in a population of animals infected at time = 0 hours. Data shown are the average of two independent experiments with 50 animals scored at each timepoint in each experiment (for a total of at least 100 animals scored at each timepoint). Error bars are SD.
(TIF)

Figure S2 **Diluted RNAi against** ***act-5*** **causes reduction in** ***YFP::ACT-5*** **expression.** *YFP::ACT-5; IFB-2::CFP* animals treated with control RNAi (A), 1:50 dilution of *act-5* RNAi (B) and 1:25 dilution of *act-5* RNAi (C). First column on left is images in the YFP channel, second column is CFP channel, third column is Nomarski bright-field and fourth column is an overlay of all three channels. Images in YFP and CFP channels were taken with same exposure time: note decreased level of *YFP::ACT-5* signal with 1:50 *act-5* RNAi and further decreased level with 1:25 *act-5* RNAi. Scale bar is 20 µm.
(TIF)

Figure S3 **Nomarski images of animals analyzed for cellular integrity.** In order to illustrate intestinal cell morphology, the same animals shown in Figure 8 A–F are shown in this figure with Nomarski bright-field imaging in A–C and Nomarski overlay with propidium iodide in red and autofluorescence in blue D–F. Upward arrows in D–F indicate lumen. Arrowheads in C indicate two examples of clusters of *N. parisii* spores, which are false-colored green. Scale bar is 20 µm.
(TIF)

Table S1 **Longitudinal studies of ACT-5 and IFB-2 changes in individual animals throughout infection.**
(XLS)

Table S2 **Analysis of** ***IFB-2::CFP*** **terminal web gap number and size in a population of animals.**
(XLS)

Table S3 **Analysis of** ***IFB-2::CFP*** **terminal web gap number and size in a longitudinal study of individual animals.**
(XLS)

Video S1 **Relocalization of intestinal actin upon infection.** Uninfected IN4000 *YFP::ACT-5* animal: note the restriction of *YFP::ACT-5* to the apical membrane. Anterior is down, pharynx is just outside of the field of view. Video plays through a Z-stack of 35 images taken every 0.5 µm in a single animal. Video starts at the plane of focus toward the outside of the animal (similar to the plane of focus in Figure 2C), and end at the plane of focus inside the animal (similar to the plane of focus in Figure 2B). See Figure 2D for more anatomical information.
(MOV)

Video S2 **Relocalization of intestinal actin upon infection.** *N. parisii* meront-infected IN4000 *YFP::ACT-5* animal. Note the extensive branched networks of ectopic *YFP::ACT-5* in basolateral regions. Lumen is slightly distended in this animal. Anterior is to the right, pharynx is just outside of the field of view. Video plays through a Z-stack of 35 images taken every 0.5 µm in a single animal. Video starts at the plane of focus toward the outside of the animal (similar to the plane of focus in Figure 2C), and end at the plane of focus inside the animal (similar to the plane of focus in Figure 2B). See Figure 2D for more anatomical information.
(MOV)
