# Supplementary figures and images for: Non-Lytic, Actin-Based Exit of Intracellular Parasites from C. elegans Intestinal Cells
Source: PLoS Pathog. 2011 Sep 15;7(9):e1002227. doi: 10.1371/journal.ppat.1002227 (PMC3174248; doi:10.1371/journal.ppat.1002227)

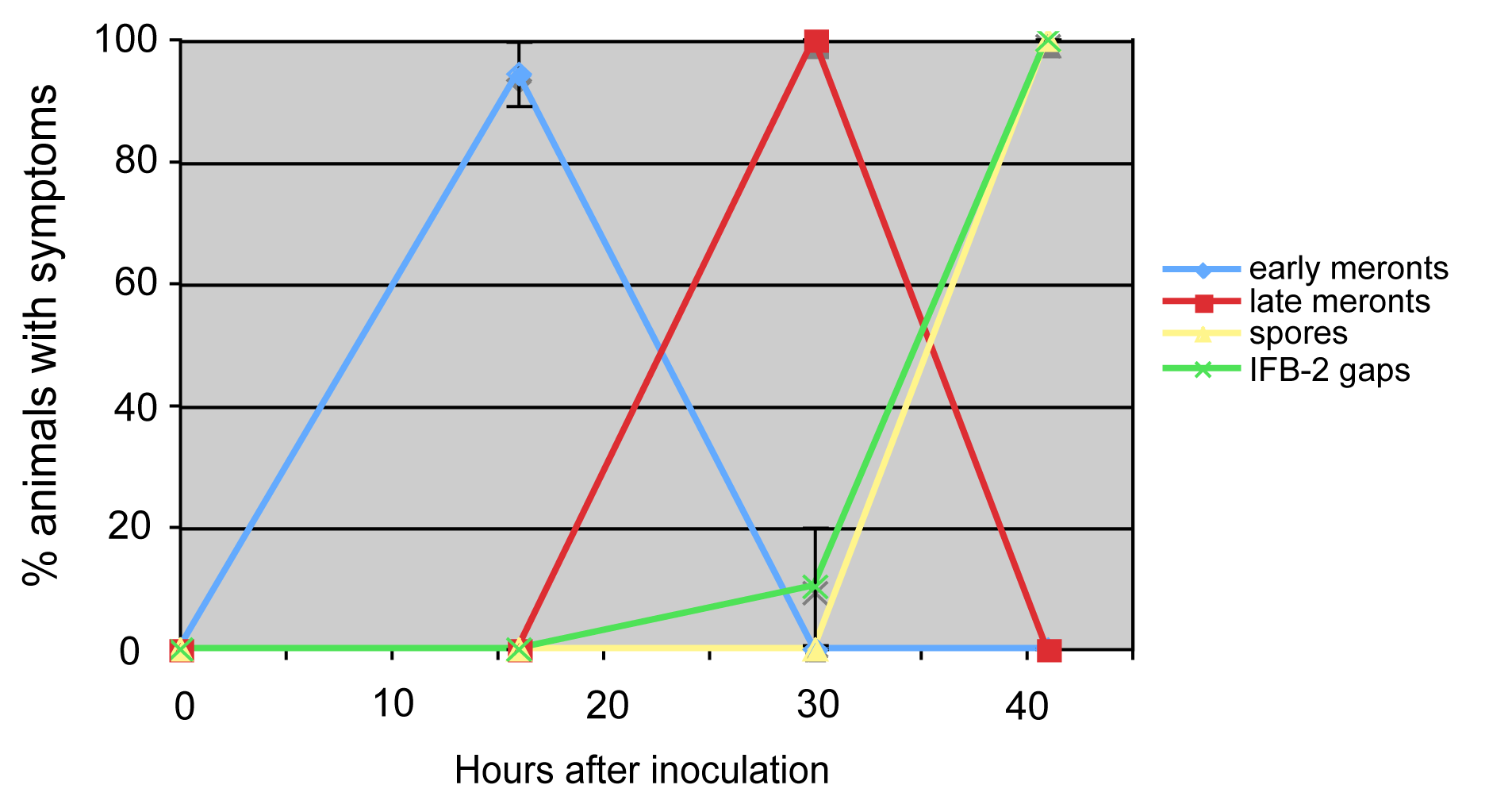

Supplement: Figure S1 — Terminal web restructuring in IFB-2::CFP single transgenic animals. Kinetics of terminal web restructuring and parasite development in a population of animals infected at time = 0 hours. Data shown are the average of two independent experiments with 50 animals scored at each timepoint in each experiment (for a total of at least 100 animals scored at each timepoint). Error bars are SD. (TIF) [file ppat.1002227.s001.tif]

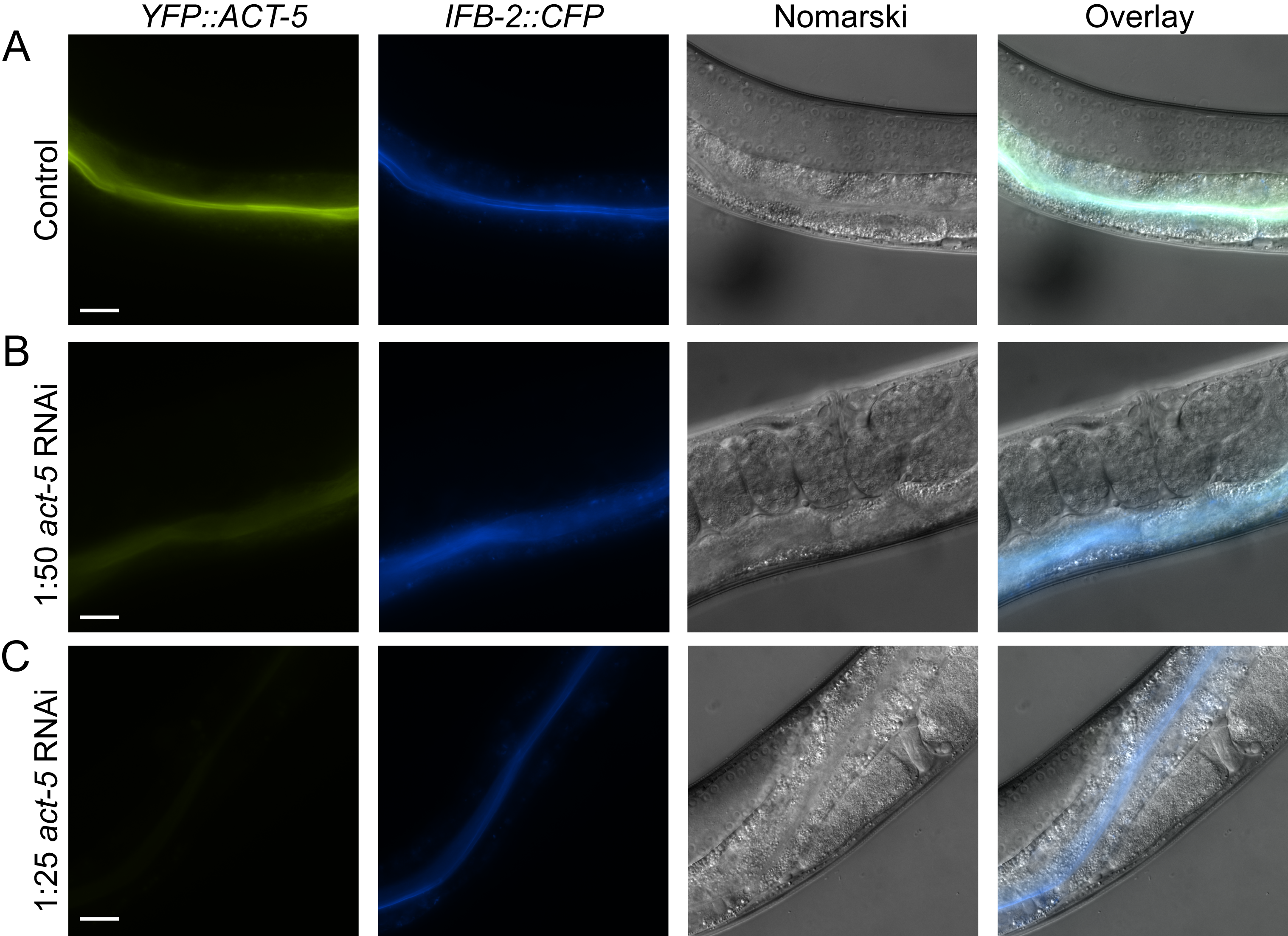

Supplement: Figure S2 — Diluted RNAi against act-5 causes reduction in YFP::ACT-5 expression. YFP::ACT-5; IFB-2::CFP animals treated with control RNAi (A), 1:50 dilution of act-5 RNAi (B) and 1:25 dilution of act-5 RNAi (C). First column on left is images in the YFP channel, second column is CFP channel, third column is Nomarski bright-field and fourth column is an overlay of all three channels. Images in YFP and CFP channels were taken with same exposure time: note decreased level of YFP::ACT-5 signal with 1:50 act-5 RNAi and further decreased level with 1:25 act-5 RNAi. Scale bar is 20 µm. (TIF) [file ppat.1002227.s002.tif]

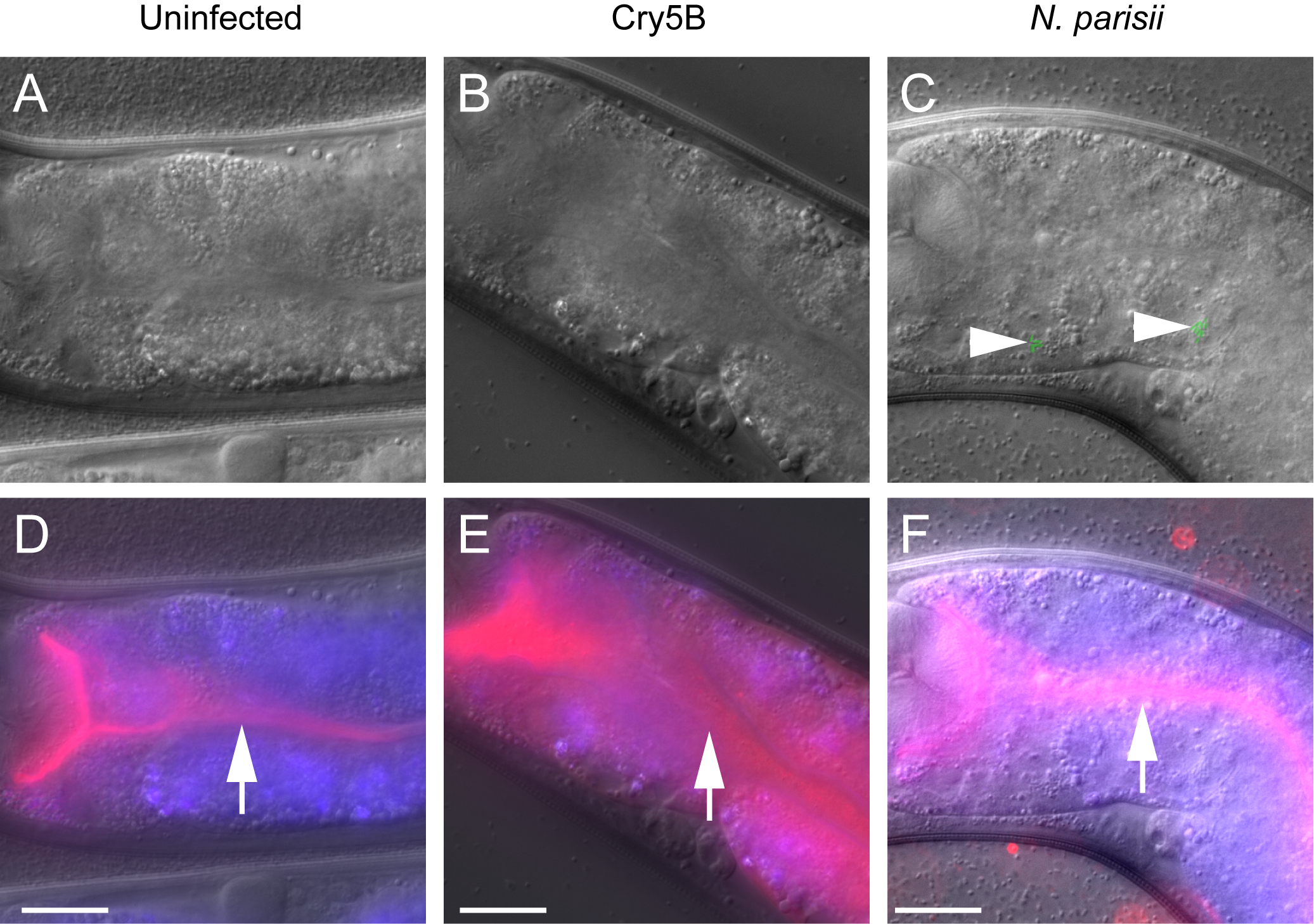

Supplement: Figure S3 — Nomarski images of animals analyzed for cellular integrity. In order to illustrate intestinal cell morphology, the same animals shown in Figure 8 A–F are shown in this figure with Nomarski bright-field imaging in A–C and Nomarski overlay with propidium iodide in red and autofluorescence in blue D–F. Upward arrows in D–F indicate lumen. Arrowheads in C indicate two examples of clusters of N. parisii spores, which are false-colored green. Scale bar is 20 µm. (TIF) [file ppat.1002227.s003.tif]
